# Common variants in the regulative regions of *GRIA1 *and *GRIA3 *receptor genes are associated with migraine susceptibility

**DOI:** 10.1186/1471-2350-11-103

**Published:** 2010-06-25

**Authors:** Daniela Formicola, Andrea Aloia, Simone Sampaolo, Olimpia Farina, Daria Diodato, Lyn R Griffiths, Fernando Gianfrancesco, Giuseppe Di Iorio, Teresa Esposito

**Affiliations:** 1 Institute of Genetics and Biophysics, Italian National Research Council, Naples, Italy; 2 Headache Service - Department of Neurological Sciences, Second University of Naples, Naples, Italy; 3 Genomics Research Centre, School of Medical Science, Griffith University, Gold Coast, Queensland, Australia

## Abstract

**Background:**

Glutamate is the principal excitatory neurotransmitter in the central nervous system which acts by the activation of either ionotropic (AMPA, NMDA and kainate receptors) or G-protein coupled metabotropic receptors. Glutamate is widely accepted to play a major role in the path physiology of migraine as implicated by data from animal and human studies. Genes involved in synthesis, metabolism and regulation of both glutamate and its receptors could be, therefore, considered as potential candidates for causing/predisposing to migraine when mutated.

**Methods:**

The association of polymorphic variants of *GRIA1*-*GRIA4 *genes which encode for the four subunits (GluR1-GluR4) of the alpha-amino-3- hydroxy-5-methyl-4-isoxazole-propionic acid (AMPA) receptor for glutamate was tested in migraineurs with and without aura (MA and MO) and healthy controls.

**Results:**

Two variants in the regulative regions of *GRIA1 *(rs2195450) and *GRIA3 *(rs3761555) genes resulted strongly associated with MA (P = 0.00002 and P = 0.0001, respectively), but not associated with MO, suggesting their role in cortical spreading depression. Whereas the rs548294 variant in *GRIA1 *gene showed association primarily with MO phenotype, supporting the hypothesis that MA and MO phenotypes could be genetically related. These variants modify binding sites for transcription factors altering the expression of *GRIA1 *and *GRIA3 *genes in different conditions.

**Conclusions:**

This study represents the first genetic evidence of a link between glutamate receptors and migraine.

## Background

Migraine is a multifactorial disorder in which genetic factors play a relevant role in both predisposing and determining underlying mechanisms. Approaches to identified genes for monogenic subtypes migraine (e.g. Familial Hemiplegic Migraine - FHM) has been successful (*CACNA1A*, *ATP1A2*, *SCN1A *genes causing FMH 1, 2 and 3 respectively) [1-4]. Conversely, finding genes for the most frequent types of migraine (with and without aura) and defining their pathogenic role has proven much more difficult [5]. However, synaptic hyper-excitability has been invocated as one major pathogenic mechanism in both common and monogenic forms of migraine, and data from animal (experimental cortical spreading depression; c-fos protein expression at the trigeminal nucleus caudalis, plasma protein extravasations at level of dura-mater circulation, electrophysiological studies) [6,7] and human (assessment of glutamate concentration in plasma, platelets and cerebrospinal fluid in migraneurs vs. control, glutamate and migraine symptoms; glutamate and sensitization) [8] studies suggest that glutamate is one of the principal factors involved.

Glutamate is the major excitatory neurotransmitter in the central nervous system which exerts this action mainly through its interaction with its ionotropic or metabotropic receptors.

In this study we investigated the association of *GRIA1, GRIA2, GRIA3 *and *GRIA4 *genes that encode for the four subunits (GluR1-GluR4) of the alpha-amino-3-hydroxy-5-methyl-4-isoxazole-propionic acid (AMPA) ionotropic receptor and their variants to migraine with and without aura (MA and MO).

This is the first study that shows a significant association between genetic variants of glutamate receptor and MA.

## Methods

### Population Collection & Phenotype

Two hundred fifty outpatients consecutively observed from January 2006 to December 2008 at the Service of Diagnosis and Therapy of Headache of the Second University, Naples and two hundred sixty healthy volunteers consecutively enrolled during the same time interval were included in the study. Cases and controls were matched for sex, came from the same small geographical area and were Caucasian. All gave informed consent to the research procedures including molecular genetic analyses. The study protocol included history, direct clinical and neurological examination, MRI with both arterial and venous angiography sequences; venous blood collection for routine blood analysis as well as DNA extraction from lymphocytes according to standard salting out procedure [9]. Migraineurs were diagnosed as having either migraine with (MA) or migraine without (MO) aura, according to the 2004 International Headache Society [10] diagnostic criteria. The study population was comprised of 244 migraineurs (M/F 56/188; age 14-64 years; MA/MO 135/109); 7 patients who resulted affected with Familial Hemiplegic Migraine were excluded from the study.

For the statistical analysis three experimental groups were considered: all migraineurs together (MA+MO= M) and MA and MO separately. Healthy control individuals (204 females and 56 males aged 21-64 years) of Caucasian origin matching for age and sex the migraineurs served as control for all three experimental groups.

### Polymorphisms selection and Genotyping

SNP selection was performed inspecting the HapMap database PhaseIII/Rel 2, Feb09 on NCBI B36 assembly, dbSNP b126 http://www.hapmap.org/index.html.en. Default parameters in Haploview were used to create haplotype blocks (95% confidence intervals) as described by Gabriel et al (2002) [11].

TagSNPs were selected at an *r*^2 ^threshold of 0.80 from all SNPs with minor allele frequency (MAF) > 0.15 allowing us to capture more than 80% of the haplotypic diversity of these genes.

#### GRIA1

**Figure 1:**
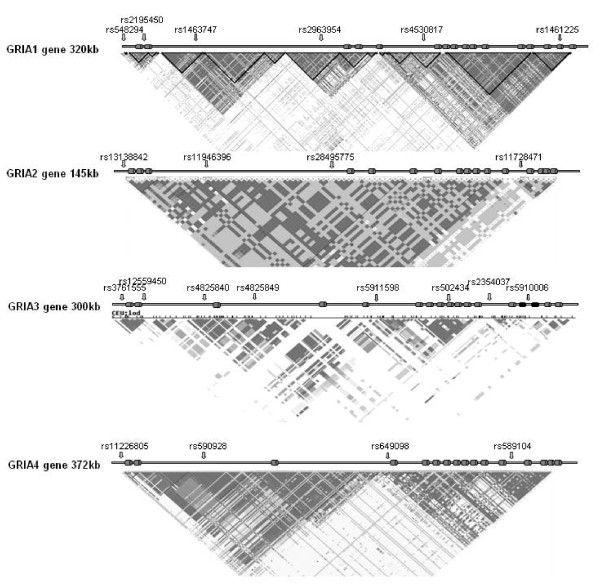
**For each gene is reported the genomic structure, the LD plot with the genotyped SNPs**. For *GRIA3 *gene the exons indicated in dark colour are alternative spliced in two different transcripts.

The gene maps on chromosome 5q33.2 and spans a genomic region of about 320 kb. After consulting the HapMap database http://www.hapmap.org/index.html.en, we identified along the 320 kb of the *GRIA1 *gene four main blocks of linkage disequilibrium (LD) with few major haplotypes. The first block included the first two *GRIA1 *exons and the 5' upstream region. The second block was in intron 2, the third block included the exons 3-5, and the remaining part of the *GRIA1 *gene and the end was in the fourth block of LD. In consideration of the extension of LD inside each block we selected two SNPs in the first block, two SNPs in the fourth block and one SNP in the second and third blocks. In particular, the SNPs chosen were: rs548294, rs2195450, rs1463747, rs2963954, rs4530817 and rs1461225 (figure 1). All SNPs were genotyped by restriction digestion using the enzymes MwoI, TaqI, BsgI, RsaI, MnlI, and HpaI respectively.

#### GRIA2

For the *GRIA2 *gene, that expands a genomic region of 143 kb on chromosome 4q32.1, two main blocks of LD were identified. We selected four SNPs (two for each block): rs13138842, rs11946396, rs28495775 and rs11728471 that were genotyped by restriction digestion using the enzymes MluI, AccII, HuncII, and HphI respectively (figure 1).

#### GRIA3

The *GRIA3 *gene spans a genomic region of about 300 kb consisting of 17 exons, and is located in Xq24-q28 region, a critical region for migraine [12]. In a previous unpublished study we completely sequenced the coding and regulative regions of the *GRIA3 *gene in ten Italian migraine patients. We detected only a synonymous sequence variation in coding region C/T N400N (rs502434), and a number of SNPs located in intronic and promoter region. Taking in account SNPs distribution and LD-plot for *GRIA3 *gene, in a first step we selected 8 SNPs located in different LD-blocks: -1952T/C SNP (rs3761555) in the promoter region, the G/A SNP rs12559450 in intron 2, the G/A SNP rs4825840 in intron 2, the C/A SNP rs4825849 in intron 3, the T/C SNP rs5911598 in intron 5, the C/T SNP N400N (rs502434) in exon 9, the C/T SNP rs2354037 in intron 12 and the T/C SNP rs5910006 in intron 14 (figure 1). In a preliminary analysis, using a smaller size cohort of migraine patients and controls (100 cases vs 100 controls) only one SNPs rs3761555 showed a trend of allelic differences between cases and controls, than was analysed in more detail. Genotyping was performed by restriction digestion using the enzyme BglII.

#### GRIA4

Also for the *GRIA4 *gene, which spans over 370 kb on chromosome 11q22.3, only two main blocks of LD were identified. We selected four SNPs (two for each block): rs11226805, rs590928, rs649098 and rs589104 that were genotyped by restriction digestion using the enzymes DdeI, Eco57I, MboI, and NlaIII respectively (figure 1).

All PCRs were performed as described in our previous work [13].

Briefly, PCR reactions (10 μl final volume) containing 2 mmol/L MgCl2, 0.5 mol/L of each primer, 200 mol/L dNTPs, 1 unit of Taq polymerase (Fermentas) and approximately 20 ng of genomic DNA were undertaken for genotyping purposes.

Thermal cycling was performed with an initial denaturation of 180 seconds at 94°C, followed by 35 cycles of 30 sec at 94°C, 30 sec at melting temperature (MT), 30 sec at 72°C, and a terminal extension of 10 min at 72°C. PCR products were then digested with restriction enzymes and analyzed by electrophoresis on 3% agarose gels. Ethidium bromide stained gels were digitally imaged and manually scored for genotypes. Table 1 shows the oligo sets and genotyping procedures for the *GRIA1 *and *GRIA3 *associated SNPs.

**Table 1:** primer sets, melting temperature (MT), PCRs size and genotyping conditions are reported for associated SNPs in *GRIA1 *and *GRIA3 *genes.

GENE	SNP	PRIMERS	MT	SIZE	ENZYME	ALLELE 1 (size)	ALLELE 2 (size)
GRIA1	rs548294	AGATGAAGAAACAGAGGTC	56°C	311 bp	MwoI	C (123/188)	T (311)
		CCCCAGGTACTATTCAAAG					
GRIA1	rs2195450	TCTAAGAGGAGGGGGCAAGG	60°C	367 bp	TaqI	G (218/149)	A (367)
		GCTTGGTAGATGGTGCTTGA					
GRIA3	rs3761555	CTGGAACAATGGAACAAAAT	55°C	272 bp	BglII	T (272)	C (111/161)
		ATAATGCTATGTCCCTGTCT					

### Association Analysis

To detect association between the tested markers and migraine, we performed chi-square (χ^2^) analysis to test for significant differences in allele and genotype frequencies in case versus control results. χ^2 ^provides the likelihood of a deviation in the distribution of the same attributes in different classes (e.g. allelic frequencies in controls versus affected subjects). If the probability (p-value) of an equal distribution between the two groups is below a determined significance level α (in percent), the statistical output will show enough significance to assume LD and therefore association. The p value of this test is asymptotically equal to the p value obtained in Armitage's trend test (ATT). ATT takes into account genotypes rather than alleles, avoiding a possible bias caused by doubling the sample size. It assumes an additive (or co-dominant) disease model where all disease alleles have equal and independent contribution to disease risk. The ATT is valid and powerful under a broad range of disease mechanisms. Second, allele and genotype frequencies, dominant and recessive genetic models as well as odds ratios were calculated to characterise the distribution of a distinct genotypes in different phenotypic subgroups of the population (using FINETTI, http://ihg.gsf.de/cgi-bin/hw/hwa1.pl). Hardy-Weinberg Equilibrium (HWE) was also calculated using FINETTI. The analysis of the statistical power was performed using the Genetic Power Calculator software http://pngu.mgh.harvard.edu/~purcell/gpc/cc2.html[14] assuming a significance level (α) of 0.05, a lifetime risk of 2, and the MAF of each SNP as calculated in our control group. In the population-based association analysis, the nominal significance threshold was set at P < 0.05 and lowered to P < 0.0017 after the multiple comparison correction of Bonferroni considering 22 SNPs, the three clinical groups analyzed, genotypes-alleles and females-males. Confidence intervals at 95% are provided for all odds-ratio (OR) values. Haplotype frequencies were calculated using the Estimation Haplotype (EH) program (Jurg Ott, Rockefeller University). A P level of less than 0.05 was considered statistically significant.

### Electrophoretic mobility shift assays

To determine whether the c.-1952T>C SNP in *GRIA3 *promoter region alters nuclear protein-DNA interactions, EMSAs was performed using radio-labelled oligonucleotide probes containing either -1952T or -1952C. Nuclear extracts were prepared from HEK 293 and SH5Y5Y cell lines as described by Granelli-Piperno et al. [15]. Binding reactions mixture contained 10 ng 32P-labeled oligonucleotide probe (10^5 ^cpm), 15 μg nuclear extract, 2 μg poly (dI-dC), 100 mM KCl, 10 mM MgCl2, 20 mM HEPES (pH 7.9), 0.2 mM EDTA, 0.5 mM dithiothreitol, 20% glycerol and protease inhibitor, in a total vol of 20 μl. To perform super shift experiments, 2 μg of the anti-HSF1 antibody (Celbio) was added to the binding reaction mixture. In competition experiments, 100-fold molar excess of cold oligonucleotides was added to the binding reaction mixture. Reaction mixtures were incubated for 30 min at room temperature, resolved on non-denaturing 5% polyacrylamide gel, dried, and exposed to autoradiography. The same assay was performed for the c.-2012C>T SNP rs548294 in *GRIA1 *promoter region and for c.+561G>A SNP rs2195450 in intron 1.

Primers used for *GRIA3 *SNP were:

GRIA3-T-EMSA-F ATGGAGACAAAAGAT**T**TCTATGAGTGGTGGGTGGG

GRIA3-T-EMSA-R TCCTCCCACCCACCACTCATAGA**A**ATCTTTTGTCT

GRIA3-C-EMSA-F ATGGAGACAAAAGAT**C**TCTATGAGTCGTGGGTGGG

GRIA3-C-EMSA-R TCCTCCCACCCACGACTCATAGA**G**ATCTTTTGTCT

Primers used for *GRIA1 *SNP rs548294 were:

GRIA1PROMF-T CAGTGCTTGCTG**T**TATTAGAGCCT

GRIA1PROMR-T TCTTAGGCTCTAATA**A**CAGCAAGC

GRIA1PROMF-C CAGTGCTTGCTG**C**TATTAGAGCCT

GRIA1PROMR-C TCTTAGGCTCTAATA**G**CAGCAAGC

Primers used for *GRIA1 *SNP rs2195450 were:

GRIA1intF-G AAAGAGACCCTC**G**AGAAGAAGGAG

GRIA1intR-G CTCACTCCTTCTTCT**C**GAGGGTCT

GRIA1intF-A AAAGAGACCCTC**A**AGAAGAAGGAG

GRIA1intR-A CTCACTCCTTCTTCT**T**GAGGGTCT

### Luciferase assays

To test the functional activity of the T>C polymorphism in the promoter region of the *GRIA3 *gene, we first identified the putative start site at -823 to the ATG, using the Promoter scan software vs2. A fragment of 1854 bp of the *GRIA3 *promoter encompassing base pairs -1411 to +325 (referred to the predicted start site) and including the T>C polymorphism was amplified from genomic DNA of migraine patients with genotype CC and controls with genotype TT. The primers used for generating this fragment were sense: 5'-GCGCGC**CTCGAG**TTTGAAGATGAGAGAACTGG-3' and antisense: 5'-GCGCGC**AAGCTT**CAAAAGAAAGAGAACGAAAG-3' which contain 5'-XhoI and 3'-HindIII restriction sites (highlighted bold sequences), respectively.

PCR products were double digested using XhoI and HindIII and then legated into a linearized pGL3-Basic vector (Promega, Madison USA) containing a Firefly Luciferase reporter gene. The inserts of the constructs were verified by sequencing before transient transfection.

Transfections were carried out using HEK293T cells. Cells were co-transfected with 1 μg of reporter plasmid and 10 ng of pRL-TK control vector (Promega, Madison USA) containing Renilla luciferase, which was used to correct for transfection efficiency. Transfection cells were harvested after 24 h and cell lysates were assayed sequentially for Firefly and Renilla luciferase activity using a dual-reporter assay system (Promega, Madison USA). Transfections were performed in quadruplicate for each plasmid construct. Changes in gene transcription were calculated relative to the corrected luciferase activity of the empty pGL3-Basic vector (control).

### Ethical approval

This research was reviewed and approved by the University of Naples Human Research Ethics Committee and all subjects participating in the study gave informed consent.

## Results

### *GRIA1 *and *GRIA3 *genes are associated with migraine

We selected SNPs for each gene (*GRIA1*-*GRIA4*) considering their distribution in LD blocks (figure 1). All variants were genotyped in a well-characterized panel of 244 cases and 260 controls (see methods). In controls, all SNPs analyzed are in Hardy-Weinberg equilibrium (HWE) (with the exception of rs2195450) and showed allele frequencies comparable to those observed in the Caucasian population and reported in the HapMap or SNP browser databases.

No significant association with the disease for any of the four SNPs in *GRIA2 *and *GRIA4 *genes tested was observed.

Significant association was observed for two SNPs located in the first block of LD of *GRIA1 *gene including the regulative region and the first two exons. Allelic and genotype frequencies distribution of the promoter -2012C/T (rs548294) marker in cases and controls showed significant association with migraine (p = 0.00009 and p = 0.0005, respectively). Stratified analyses of migraine subtypes were also undertaken indicating association primarily attributed to MO subtype group (P = 0.0003) and particularly in females (P = 0.0007) (Table 2). These differences remained statistically significant when Bonferroni correction for multiple comparisons was applied (Migraine all: alleles p = 0.002; genotypes p = 0.01; MO alleles: p = 0.008; genotypes p = 0.03; Females all: alleles p = 0.02; genotypes p = 0.05) (Table 2).

**Table 2:** Allele and genotype frequencies of *GRIA1 *and *GRIA3 *polymorphisms in Italian patients and controls and association results

Gene	SNP	SAMPLE	N	MAF	SP	MAJOR HOMO	HET	MINOR HOMO	ALLELE P-VALUE	11 vs 12	GENOTYPE P-VALUE 11 vs 22	11 vs 12+22	ARMITAGE'S TEST -P VALUE
***GRIA1***	RS548294 C/T	CONTROLS	260	34%		125 (48,1%)	102 (39,2%)	33 (12,7%)					
		CONT.FEMALE	204	32,3%		98 (48%)	80 (39,2%)	26 (12,8%)					
		CONT. MALE	56	32,1%		27 (48,2%)	22 (39,3%)	7 (12,5%)					
		MIGRAINE ALL	244	44,3%	97%	80 (32,8%)	112 (45,9%)	52 (21,3%)	0,00009 (0,002*) (OR 1,7/CI 1,2-2,1)	**0,006 **(NS*) (OR 1,7/CI 1,1-2,5)	0,0005 (0,01*) (OR 2,4/CI 1,5-4,1)	0,0005 (0,01*) (OR 1,9/CI 1,3-2,7)	0,0002 (0,005*)
		MA	135	42,6%	80%	52 (38,5%)	51 (37,8%)	32 (23,7%)	**0,004 **(NS*) (OR 1,6/CI 1,1-2,1)	0,4 (NS*)	**0,004 **(NS*) (OR 2,3/CI 1,3-4,1)	0,07 (NS*)	**0,007 **(NS*)
		MO	109	46,3%	78%	28 (25,7%)	61 (56%)	20 (18,3%)	0,0003 (0,008*) (OR 1,8/CI 1,3-2,5)	0,0001 (0,003*) (OR 2,6/CI 1,6-4,5)	**0,003 **(NS*) (OR 2,7/CI 1,3-5,3)	**0,00007 **(0,002*) (OR 2,7/CI 1,6-4,3)	0,0004 (0,01*)
		MIG. FEMALE	188	44,1%	90%	63 (33,5%)	84 (44,7%)	41 (21,5%)	0,0007 (0,02*) (OR 1,6/CI 1,2-2,2)	**0,02 **(NS*) (OR 1,6/CI 1,0-2,5)	0,002 (0,05*) (OR 2,4/CI 1,3-4,4)	**0,003 **(NS*) (OR 1,8/CI 1,2-2,7)	0,001 (0,03*)
		MIG. MALE	56	41%	38%	19 (34%)	28 (50%)	9 (16%)	0,1	0,1	0,3	0,1	0,1
	RS2195450 G/A	CONTROLS	260	34,2%		128 (49,2%)	86 (33%)	46 (17,8%)					
		CONT. FEMALE	204	34,5%		95 (46,6%)	77 (37,7%)	32 (15,7%)					
		CONT. MALE	56	33%		30 (53,6%)	15 (26,7%)	11 (19,6%)					
		MIGRAINE ALL	244	45,5%	97%	98 (40,1%)	70 (28,7%)	76 (31,2%)	0,0002 (0,005*) (OR 1,6/CI 1,2-2,0)	0,7	0,0007 (0,02*) (OR 2,1/CI 1,3-3,3)	**0,04 **(NS*) (OR 1,4/CI 1-2)	0,001 (0,03*)
		MA	135	50%	80%	51 (38%)	33 (24%)	51 (38%)	0,00002 (0,0005*) (OR 1,9/CI 1,4-2,5)	0,8	0,00008 (0,002*) (OR 2,7/CI 1,6-4,6)	**0,03 **(NS*) (OR 1,6/CI 1,0-2,4)	0,0002 (0,005*)
		MO	109	39%	78%	47 (43,2%)	39 (35,7%)	23 (21,1%)	0,2	0,4	0,3	0,2	0,2
		MIG. FEMALE	188	42,3%	90%	83 (44,1%)	51 (27,1%)	54 (28,8%)	**0,02 **(NS*) (OR 1,3/CI 1-1,8)	0,2	**0,01 **(NS*) (OR 1,9/CI 1,1-3,2)	0,6	**0,05 **(NS*)
		MIG. MALE	56	53,5%	38%	17 (30,3%)	18 (32,1%)	21 (37,5%)	0,001 (0,03*) (OR 2,3/CI 1,3-4)	0,1	**0,01 **(NS*) (OR 3,3/CI 1,3-8,6)	**0,01 **(NS*) (OR 2,6/CI 1,2-5,7)	**0,008 **(NS*)
***GRIA3***	RS3761555 T/C	CONTROLS	260	22,3%		170 (65,3%)	64 (24,6%)	26 (10,1%)					
		CONT.FEMALE	204	23,5%		124 (60,7%)	64 (31,3%)	16 (8%)					
		CONT. MALE	56	17,9%		46 (82,1%)		10 (17,9%)					
		MIG. FEM.ALL	188	34,6%	93%	83 (44,1%)	80 (42,5%)	25 (13,4%)	0,0006 (0,01*) (OR 1,7/CI 1,2-2,3)	**0,004 (**NS*) (OR 1,8/CI 1,2-2,8)	**0,01 (**NS*) (OR 2,3/CI 1,1-4,6)	0,0009 (0,02*) (OR 1,9/CI 1,3-2,9)	0,001 (0,003*)
		MA-FEMALE	112	38%	84%	44 (39,3%)	51 (45,5%)	17 (15,2%)	0,0001 (0,003*) (OR 2/CI 1,4-2,8)	**0,001 **(0,03*) (OR 2,3/CI 1,3-3,7)	**0,003 (**NS*) (OR 3/CI 1,4-6,4)	0,0002 (0,005*) (OR 2,4/CI 1,5-3,8)	0,0002 (0,005*)
		MO-FEMALE	76	30%	73%	38 (50%)	30 (39,5%)	8 (10,5%)	0,1	0,1	0,2	0,1	0,1
		MIG. MALE	56		40%	36 (64,5%)		20 (35,5%)	**0,03 **(NS*) (OR 2,46/CI 1,07-5,6)				

p-values for allele and genotype distributions are showed. Minor allele frequency is indicated as MAF. To assess the dosage effect of possessing zero, one or two copies of the risk allele (i.e. an additive effect), the Armitage test for linear trend in proportions was performed on the genotype frequency data. Genetic risk magnitudes (effect size) were estimated by calculating odds ratio (ORs) with 95% confidence intervals (95% CI). All statistical tests were two-tailed. Statistical significance was defined as a p value lower than 0.05. NS, not significant after Bonferroni correction, * Pvalues after Bonferroni correction. The percentages of genotype and allele frequency are in parentheses. The Pvalues less than 0.05 are in bold. Statistical power (SP) was calculated for all groups and subgroups analyzed.

The analysis of the +561 G>A SNP (rs2195450) also showed significant association in both allelic and genotype distributions (p = 0.0002 and p = 0.0007, respectively) (p = 0.005 and p = 0.02 after Bonferroni corrections). Stratified analyses of migraine subtypes showed a specific association with MA subtype group for both allelic and genotypic frequencies (p = 0.00002 and p = 0.00008) (p = 0.0005 and p = 0.002 after Bonferroni corrections) (Table 2). However, the SNP rs2195450, was the only SNP found in Hardy-Weinberg disequilibrium (DHW) both in controls and in patients (p < 0.05).

Regarding *GRIA3 *gene, we obtained statistically significant evidence for an association to migraine phenotype for the -1952T/C variant (rs3761555) located in the regulative region of the gene (allele C frequencies: controls-migraineurs = 22% vs. 34%) (Table 2). In consideration of the X chromosomal localization of *GRIA3 *gene, we analyzed the data by gender, showing significant associations in females for both allelic and genotype distributions (p = 0.0006 and p = 0.0009, respectively). Stratified analyses of migraine subtypes showed a specific association with the female MA subtype group for either allelic or genotypic frequencies (p = 0.0001 and p = 0.0002, respectively) (Table 2). These differences remained statistically significant after Bonferroni correction (alleles p = 0.003; genotypes p = 0.005) (Table 2). The male group was not analyzed independently due to its limited size (n = 56). The statistical power for the three associated SNPs in *GRIA1 *and *GRIA3 *genes was 97% for rs548294, 97% for rs2195450 and 93% for rs3761555. When we calculated the statistical power for subgroups (MA, MO, females, males) it remained high in MA and females, but it decreased for MO and males subgroup due to their limited size. We analyzed the distribution of the haplotypes formed by the associated SNPs in *GRIA1 *gene in cases and controls. Haplotype frequencies (calculated using the EH program) were significantly different in cases and controls (chi-square = 12.72, df = 3, p-value 0.005). Haplotype-specific testing showed that the putative risk haplotype T_A (rs548294- rs2195450) was more frequent in cases than in controls, resulting in p-value of 0.002 and an OR of 2.1. Conversely, the putative protective haplotype C_G (rs548294- rs2195450) was more frequent in controls than in cases resulting in a p-value of 0.00001 and an OR of 0.4 (Table 3).

**Table 3:** Frequency of common *GRIA1 *haplotypes in migraine patients compared with healthy controls.

HAPLOTYPE	C**ASES N = 452**	PERCENTAGE	CONTROLS N = 504	PERCENTAGE	P-VALUE	ODDS RATIO	95% CONFIDENCE INTERVAL
C__A	149	33%	131	26%	0.0180	1.40	1.06-1.85
C__G	99	22%	207	41%	0.00001	0.40	0.30-0.54
T__A	59	13%	35	7%	0.0015	2.10	1.30-3.12
T__G	145	32%	131	26%	0.0381	1.34	1.02-1.78

Possible additive interactions of the two most associated SNPs in *GRIA1 *(rs548294) and *GRIA3 *(rs3761555) genes were evaluated in those subjects who had both SNPs characterization. *GRIA1 *SNP rs2195450 was not analyzed because it was in HWD. In Migraine patients, the p-value for the sole risk allele of the *GRIA1 *gene (TT/TT, CT/TT genotypes) was 0.03, OR 1.68 (CI = 1.02-2.77); the p-value for the sole risk allele of the *GRIA3 *gene (CC/TC, CC/CC genotypes) was 0.005, 2.28 (CI = 1.27-4.11) and the p-value for the combined two risk alleles (TT/TC, TT/CC, CT/TC, CT/CC genotypes) was 0.00001, OR 3.35 (CI = 1.96-5.74) (Table 4).

**Table 4:** Combined genotypic frequencies of *GRIA1 *(CT) and *GRIA3 *(TC)

	*C**ONTROLS N* = 260	CASES *N* = 244	*P-VALUE*	*OR*	*95% CI*
TWO RISK ALLELES	6+3+31+9 (19%)	24+6+29+22 (33%)	0.00001	3.35	1.96-5.74
ONE RISK ALLELE (GRIA1)	26+74 (39%)	20+63 (34%)	0.03	1.68	1.02-2.77
ONE RISK ALLELE (GRIA3)	26+14 (15%)	27+18 (19%)	0.005	2.28	1.27-4.11
NO RISK ALLELE	71 (27%)	35 (14%)			

Genotypes for two risk alleles were: TT/TC + TT/CC + CT/TC + CT/CC; one risk allele (*GRIA1*): TT/TT + CT/TT; one risk allele (*GRIA3*): CC/TC + CC/CC; no risk allele: CC/TT.

### -1952 T>C variant in *GRIA3 *regulative region have functional significance

**Figure 2:**
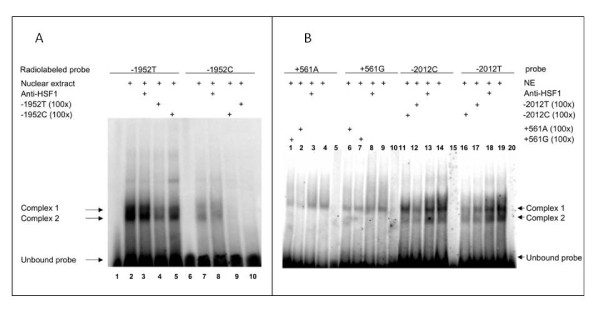
**A, electrophoretic mobility shift assay (EMSA) with radiolabeled either -1952T or -1952C probe and HEK-293 cell nuclear extracts (NE) and B, EMSA assays with radiolabeled either -2012T/-2012C and +561G/+561A probes and HEK-293 cell NE**. **A**. Lanes 1 and 6, mobility of the labelled probes without NE; lanes 2 and 7, mobility of the labelled probes with NE in the absence of competitor. A specific nuclear protein binding can be almost completely abolished both by 100-fold unlabeled -1952T but not with -1952C probe (lanes 4 and 5; lanes 9 and 10). Super shift assays incubating with anti-HSF1 antibody did not show any super shifted protein complex (lanes 3 and 8). **B**. Lanes 5, 10, 15 and 20, mobility of the labelled probes without NE; lanes 4, 9, 14, and 19, mobility of the labelled probes with NE in the absence of competitor. Competition assays with 100-fold unlabeled probes (lanes 1 and 2; lanes 6 and 7; lanes 11 and 12; lanes 16 and 17). Super shift assays incubating with anti-HSF1 antibody did not show any super shifted protein complex (lanes 3, 8, 13 and 18).

Bioinformatics predictions by TRANSFAC [16] showed that the -1952T>C associated variant could affect the putative transcription factor binding sites HSF (Heat Shock Factor) and CdxA. To determine whether the c.-1952T>C SNP alters nuclear protein-DNA interactions, EMSAs were performed using radio-labelled oligonucleotide probes containing either -1952T or -1952C (see materials and methods). Two distinct complexes were revealed on incubation of the probes with nuclear extract from unstimulated HEK293T cells (figure 2A) and the -1952T probe showed stronger DNA protein-binding activity compared with the -1952C probe. EMSA assay was also performed using nuclear extract from neuroblastoma cell line SH5Y5Y and obtained the same results (data not shown). To further assess the binding specificity and the differences in binding affinity between the T and C allele, competition assay was performed with unlabeled -1952T or -1952C oligonucleotide. The results revealed that unlabeled -1952T oligonucleotide but not -1952C with 100- fold molar excess fully blocked the binding of the radiolabeled -1952T probe or -1952C probe with nuclear protein(s). Moreover, to determine if HSF1 is one of the transcription factors bound to the complexes, anti-HSF1 antibody was used in super shift assays. In these experimental conditions none of the complexes was specific for HSF1 (figure 2A). These data suggest that the -1952T>C polymorphism can affect the binding affinity of the *GRIA3 *promoter with several transcription factor(s) and the variant T allele has a stronger binding strength compared with the C allele. To test the functional effect of the -1952 T>C variant we performed the luciferase assay using the promoter region of *GRIA3 *gene.

**Figure 3:**
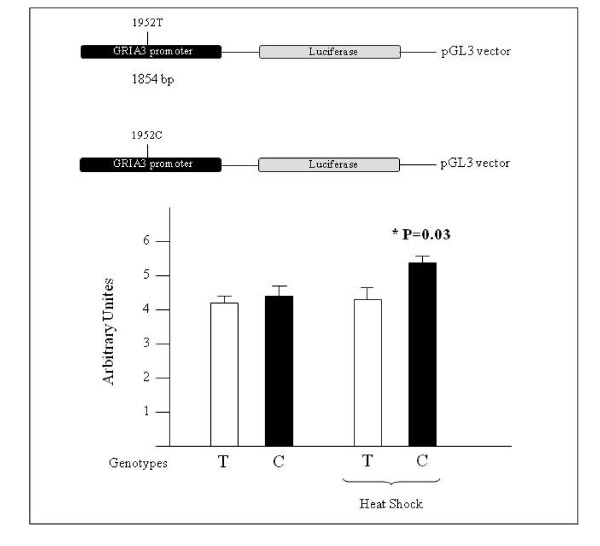
**Luciferase assay for *GRIA3 *promoter activity**. Under heat shock condition the luciferase activity generated by the C construct was 1.3-fold greater than that generated by the T construct (p < 0.05 using t-test calculation).

When HEK293T cells were transfected with the -1411 to +325 region, the luciferase activity generated by the C construct was similar to that generated by the T construct but interestingly, under heat shock condition (at 40°C for 1 h) the luciferase activity generated by the C construct was 1.3-fold greater than that generated by the T construct (figure 3).

### Functional analysis of the associated *GRIA1 *variants

Bioinformatics predictions did not show any clear alteration of transcription factor binding sites both for SNP -2012C>T and +561G>A. To determine whether these variations could alter nuclear protein-DNA interactions we performed EMSAs assays using radiolabelled oligonucleotide probes containing either -2012C or -2012T and +561G or +561A (see materials and methods) (figure 2B). No binding affinity was revealed for both +561G and +561A alleles. In contrast, two distinct complexes were revealed on incubation of the probes -2012T and -2012C with nuclear extract from unstimulated HEK293T cells and in particular, the -2012T probe showed stronger DNA protein-binding activity compared with the -2012C probe (figure 2B). The binding specificity and the differences in binding affinity between the T and C allele was also demonstrated in competition assay using unlabeled -2012T or -2012C oligonucleotide. The results revealed that unlabeled -2012T oligonucleotide but not -2012C with 100- fold molar excess fully blocked the binding of the radiolabeled -2012T probe or -2012C probe with nuclear protein(s) (figure 2B).

### *GRIAs *comparative analysis

**Figure 4:**
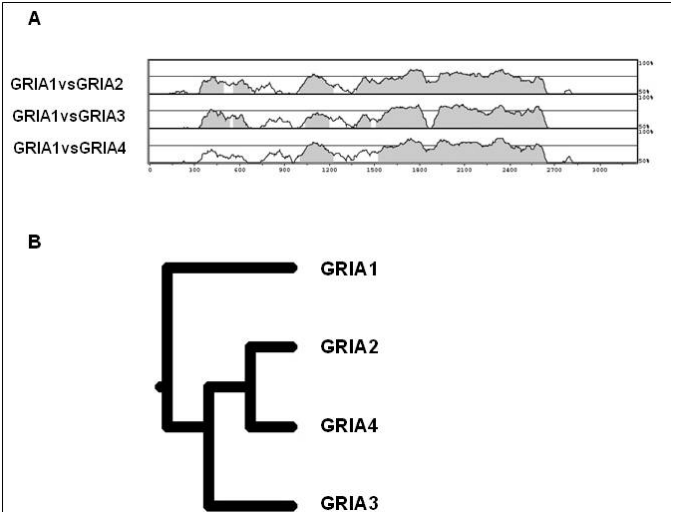
**Comparative analysis of *GRIA1-GRIA4 *genes**. A: comparison of *GRIA1 *coding region versus *GRIA2*, *GRIA3 *and *GRIA4 *cDNAs. B: phylogenetic tree.

The identification of associated variants in the promoter regions of both *GRIA1 *and *GRIA3 *genes and their evidence for functional roles, suggested us to investigate about the evolutive conservation of this class of genes. We selected from databases the full-length sequences of the transcripts of *GRIA1-GRIA4 *genes and the regulative regions at the 5' of each gene. To postulate a coordinate regulation of these genes, all sequences were compared using the vista software and the results suggest that *GRIA1 *seems be the ancestral gene and is more similar to *GRIA3 *gene as for other genes (*GRIA2 *and *GRIA4*) (figure 4).

## Discussion

Glutamate is a major excitatory neurotransmitter in the central nervous system, thought to be widely involved in migraine mechanisms. Indeed, migraine brain nociceptive centres, including the trigeminal ganglion, trigeminal nucleus caudalis, and thalamus, contain glutamate-positive neurons, and glutamate activates the trigeminal nucleus caudalis. Glutamate is implicated in cortical spreading depression (CSD), trigemino-vascular activation, and central sensitization [17]. These observations argue for a strong link between migraine and the glutamatergic system. A link that is important to further characterization and understanding of migraine mechanisms in order to deliver effective therapies.

In this study we have investigated the role of glutamate receptors, ionotropic, AMPA (*GRIA1, GRIA2 GRIA3 *and *GRIA4 *genes) and their variants in MA and MO. We found two variants in *GRIA1 *gene (rs548294 and rs2195450) and one variant in *GRIA3 *gene (rs3761555), all located in the regulative region of the genes, to be associated with the migraine phenotype in an Italian population. Interestingly, both the *GRIA1 *and *GRIA3 *variants were specifically associated with MA suggesting a role of these genes in CSD, which is retained to be the path physiological mechanism-underlying aura. However, to date we cannot exclude that these variant can also be associated to MO phenotype due to limited size of MO subgroup. In fact, the rs548294 SNP in GRIA1 gene was primarily associated with MO rather than MA. Although MO and MA are separate categories according to the ICHD-II criteria these two subtypes of migraine share some path physiological mechanisms [18]. Several studies support that the same SNP or different SNPs in the same gene could be associated with both MO and MA phenotypes [5].

We analyzed the distribution of the haplotypes formed by the associated SNPs in *GRIA1 *gene in cases and controls. Haplotype-specific testing showed that the putative risk haplotype T_A was more frequent in cases than in controls (13% and 7%, respectively, P = 0.001) and was associated with increased risk of migraine (OR = 2,10; 95% CI (1.30- 3,12)) compared with the risk of the two alleles taken separately. Furthermore, our analysis of the combined effects of the two SNPs in *GRIA1 *(rs548294) and *GRIA3 *(rs3761555) genes not supported a synergy of the two SNPs in the genetic susceptibility to migraine. Indeed, in migraine patients, the combined two risk allele genotypes exhibited an OR that was more or less the sum of the ORs of each risk allele taken separately, suggesting that, when both risk alleles are present, the risk to develop migraine is merely the sum of the effect carried by each single risk allele.

Although our data need to be confirmed in more numerous series as well as in populations of different origin, the identification of a statistically significant genetic link between glutamate receptor genes and MA needs to be highlighted. A growing number of scientific report based on preclinical and clinical data argue strongly for a role of glutamatergic receptor activation in migraine and in particular in CSD [19]. Despite the remarkable advancing in the pharmacotherapy, there is still a tremendous need for the research of more effective treatments. Glutamate receptors antagonists have been recently proposed as major target addressed for the treatment of migraine. Glutamate receptors indeed represent a promising target for a valuable, non-vasoactive oriented approach to the treatment of migraine [20].

The SNP rs2195450, which shows the strongest association, is in Hardy-Weinberg disequilibrium (DHW) both in controls and in patients. DHW may be due to several factors including genotyping errors, inappropriate population stratification and selection, inbreeding, presence of a causative allele or simply the chance. We can exclude with high confidence that DHW as well as patient-control differences could be due to genotyping errors. For all SNPs, about 20 subjects were randomly selected and sequenced; for rs2195450 the sample was increased to 100 subjects. The original classification was confirmed in all samples. Moreover, for the autosome genes, the presence of DHW in different direction in controls and in patients, as observed for rs2195450, is compatible with the segregation of a causative variant according to a dominant model [21]. However, we cannot exclude that DHW is due to other confounding factors, and then the association of this SNP must be considered with prudence.

Further remarkable in our cohort is that *GRIA1 *and *GRIA3 *variants are associated to migraine in female but not in male subjects. To date we cannot assert that AMPA receptor genes and genetic variations within them may act differently in the two sexes, in light of the small sample size of case males that do not provide enough statistical power to detect association.

However, the underlying mechanism by which these variants confer susceptibility to migraine remains unclear. Bioinformatics predictions showed that the C variant of *GRIA3 *gene could affect putative binding sites for transcription factors altering the consensus sequence. We strengthened this hypothesis by transfecting the HEK293T cells with a promoter region of *GRIA3 *gene. It should be highlighted that, under stress condition the luciferase activity generated by the C construct (associated allele) was 1.3-fold greater than that generated by the T construct. Moreover, EMSA assays suggest that the -1952T>C polymorphism can affect the binding affinity of the *GRIA3 *promoter with transcription factor(s) and the variant T allele has a stronger binding strength compared with the C allele. Considering that the T-allele displays lower promoter transcription activity, we infer that the nuclear factor(s) bound to the region covering the -1952T>C polymorphism may function as negative regulator(s) for *GRIA3 *transcription. Therefore, the -1952T>C polymorphism may affect the *GRIA3 *promoter binding affinity with nuclear proteins and in turn the *GRIA3 *expression, which consequently modulates the individual's susceptibility to migraine. Future genetic and functional studies are necessary to further elucidate the role of *GRIA3 *in migraine susceptibility. Preliminary results using an Australian case-control panel indicate that the same -1952T>C polymorphism showed a trend of association with migraine (p = 0.04) (L. Griffiths personal communication).

More complex is the case of *GRIA1 *gene where only the -2012C>T variant seems to affect the binding affinity of the *GRIA1 *promoter with transcription factor(s) and the variant T allele has a stronger binding strength compared with the C allele. Considering that the T-allele is also the risk allele we can infer that nuclear factor/s bound to the region covering the -2012C>T polymorphism may function as positive regulator(s) for *GRIA1 *transcription.

## Conclusions

These results indicate an involvement of *GRIA1 *and *GRIA3 *genes in the susceptibility to migraine with aura and encourage replication in other data sets. Moreover, functional investigations to explain the role of glutamate receptors variants in MA might be useful.

## Competing interests

The authors declare that they have no competing interests.

## Authors' contributions

DF, AA, FG and TE were responsible for undertaking all the experiments and the analysis of data. SS, OF DD and GDI were responsible for patients and controls collection. LRG revised the manuscript. All authors read and approved the final manuscript.

## Pre-publication history

The pre-publication history for this paper can be accessed here:

http://www.biomedcentral.com/1471-2350/11/103/prepub
